# Numerical analyses for three-dimensional face stability of circular tunnels in purely cohesive soils with linearly increasing strength

**DOI:** 10.1038/s41598-023-49065-6

**Published:** 2024-03-13

**Authors:** Bing Huang, Daidai Yu, Yinting Zhao, Junfeng Zhu

**Affiliations:** 1 Sichuan Expressway Construction and Development Group Co., Ltd, Chengdu, 610031 China; 2 Shudao Investment Group Co. LTD., Chengdu, 610031 China

**Keywords:** Engineering, Civil engineering, Geology

## Abstract

The tunnel face stability in purely cohesive soils with linearly increasing strength was investigated using three-dimensional finite element limit analysis (FELA). Both the collapse (active failure) and blow-out (passive failure) of the tunnel face were considered in the analysis. The rigorous upper bound (UB) and lower bound (LB) solutions of the load factor were calculated with a wide range of ground conditions to cover a broad scope of practical application. The results showed that the whole surface of the face is at failure in the collapse case; while in the blow-out case, there exists a gradual evolution process from partial failure to global failure within the tunnel face with increasing buried depth. Later, based on 960 finite element limit analysis results, a series of practical equations were proposed for tunnel face stability analysis in purely cohesive soils. These equations can be employed to quickly calculate the UB and LB solutions of the limit support pressure and the stability number of a tunnel face in both the collapse and blow-out cases. Finally, the calculation results from these equations were compared with those from previous studies in detail. The comparisons showed that the proposed equations make an improvement over existing methods and can be used as an efficient tool in practical engineering.

## Introduction

In shield tunnelling, one of the most crucial issues is to prevent the tunnel face from failure. Specifically, the collapse (i.e., active failure) of the tunnel face occurs when the support pressure is not sufficient to prevent the movement of the soils toward the tunnel. The blow-out (i.e., passive failure) of the tunnel face occurs when the support pressure is high enough to push the soils toward the ground surface^1^. Therefore, the determination of reasonable support pressure is a crucial problem in practical engineering. To solve this problem, a series of research have been conducted to investigate the tunnel face stability in frictional, frictional-cohesive, and purely cohesive soils, which can be mainly divided into three aspects: experimental, numerical, and theoretical methods.

In frictional and frictional-cohesive soils, several centrifuge model tests^2–6^, and 1 g model tests^7–9^ have been performed to study the limit support pressure and failure mechanism of the tunnel face. The test results revealed the failure mechanism and provided a reference for the verification of numerical and theoretical methods. Over the last decades, numerical simulations have become a common tool. Many numerical models have been established to assess the tunnel face stability using the finite element method (FEM)^10,11^, finite difference method (FDM)^12,13^, discrete element method (DEM)^14,15^ and finite element limit analysis (FELA)^16,17^. The failure mechanism of the tunnel face was investigated in detail, and a series of equations to assess the tunnel face stability were proposed. However, the numerical methods were time-consuming, therefore, many analytical models have been developed for practitioners to efficiently analyze the tunnel face stability. Horn^18^ proposed the wedge-silo model, and the first practical application of this model was conducted by Jancsecz and Steiner^19^ based on the limit equilibrium method. Later, this model was improved to be more accurate^20–22^ and modified to consider the effects of the heterogeneity of the ground^23^, reinforcements^6,24,25^, unsupported span^26^ and seepage flow^27^. However, even the wedge-silo model has been widely employed owing to its simplicity, the necessary assumptions (especially the simplified failure mechanism and the lateral pressure coefficient) make this model not rigorous enough. Another commonly used method in tunnel face stability analysis is the limit analysis method^28^ (including upper and lower bounds theorems), which is more rigorous than the limit equilibrium method. Based on the upper bound theorem of limit analysis, a series of translational rigid-block models^29–32^ and rotational rigid-block models^33,34^ have been established. The results from these analytical models, especially those established in the last decade, agree well with those from experimental tests and numerical simulations, which indicates that the failure mechanism employ in these models is reasonable.

In purely cohesive soils, experimental tests^35–39^ also have played essential roles in tunnel face stability study. The results showed that compared with the failure mechanism of the tunnel face in frictional and frictional-cohesive soils, the failure mechanism in purely cohesive soils do not have apparent localized shear band, and the soil moves more like a ‘flow’ rather than a rigid block. This conclusion also indicated that the rigid-block models that already exist for frictional and frictional-cohesive soils maybe not suitable for purely cohesive soil. In terms of numerical simulations, a series of numerical models have been established to study this problem^40–43^. It is worth noting that the equations proposed by Ukritchon et al.^41^ based on FEM results provide the optimal solution so far. However, in FEM, the limit state is obtained by the so-called stress-controlled approach instead of rigorous analysis. Besides, Ukritchon et al.^41^ only considered the collapse case and neglected the blow-out case. In terms of theoretical methods, Broms and Bennermark^38^ defined the stability number *N* based on field observation data collected during tests, and *N* has been widely employed in practical applications. Davis et al.^44^ first proposed upper and lower bound solutions for tunnel face stability analysis in undrained clay. Ellstein^45^ obtained an analytic solution employing the limit equilibrium method, and the calculation results were in close agreement with the experimental results reported by Kimura and Mair^39^. Klar et al.^46^ established two new upper bound solutions for 2D and 3D tunnel face stability analysis based on a continuous deformation of the soil. Later, Mollon et al.^1^ suggested two continuous velocity fields for the collapse and blow-out of a pressurized tunnel face in purely cohesive soils, and these velocity fields significantly improve the best existing upper bound solutions. More recently, this failure mechanism was extended for tunnel face stability analysis in nonhomogeneous undrained clay^47,48^, horseshoe-shaped shield tunnels^49,50^, and volume loss prediction for tunnel face advancement^51^. However, there are still some non-ignorable differences between the results calculated by the most advanced analytical models and those from numerical simulations^1,48,50^, which indicates that the failure mechanism employed in these analytical models need to be further improved. Besides, the partial failure mechanism in the blow-out case reported by several authors^12,52,53^ was neglected in these analytical models.

To solve these issues, this paper aims at proposing a quick and accurate method for tunnel face stability analysis in purely cohesive soils in both the collapse and blow-out cases. “Problem definition and method of analysis” describes the problem of the tunnel face stability and introduces the FELA. “Results and discussions” plots and discusses the calculation results of the load factor and failure mechanism. “New design equations” proposes a series of equations for tunnel face stability analysis in purely cohesive soils. “Comparisons with previous studies” compares the calculation results of the proposed equations with those from the existing methods.

## Problem definition and method of analysis

**Figure 1:**
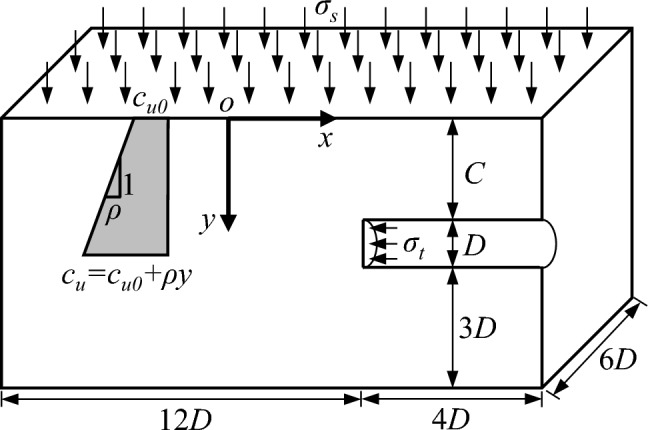
Problem definition of the 3D tunnel face stability in purely cohesive soils.

The problem definition of the present study is shown in Fig. 1, and only half of a tunnel is considered due to the problem symmetry. The tunnel has a diameter *D* and a cover depth *C* in purely cohesive soils. The soil is modeled as an elastic-perfectly plastic Tresca material. The undrained cohesion *c*_*u*_ of soil is linearly increasing with depth *y*, i.e., *c*_*u*_ = *c*_*u0*_ + *ρy*, where *c*_*u0*_ is the undrained cohesion of soil at the ground surface, and *ρ* is the variation gradient of undrained cohesion. Besides, the tunnel face is applied by a uniform support pressure *σ*_*t*_, and the ground surface is loaded by a uniform surcharge *σ*_*s*_.

Most of the available 3D numerical simulations such as finite element method (FEM), finite difference method (FDM) and discrete element method (DEM) are considered very time consuming for simulating the failure of a 3D tunnel face. The new development of 3D FELA technique is a powerful and efficient tool in calculating the rigorous upper bound (UB) and lower bound (LB) solutions of stability problems in geotechnical engineering. This numerical limit analysis method assumes a perfectly plastic material with associated flow rule, and combines the classical plasticity theorems, finite element method and mathematical optimization method^54^. In addition, the FELA does not need to make any assumptions about the failure mechanism, which allows FELA to get a more rigorous result than analytical limit analysis methods. In this work, the advanced three-dimensional commercial FELA software OptumG3^55^ was employed to calculate the rigorous UB and LB solutions of the limit collapse and blow-out pressures *σ*_*t*_ of a tunnel face.

**Figure 2:**
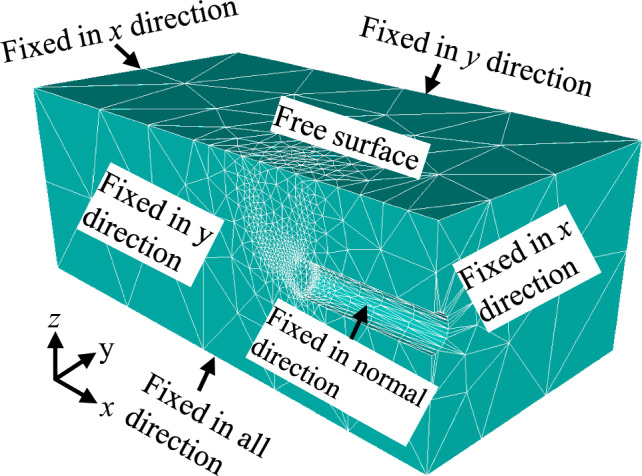
Adaptive mesh and boundary conditions (*C*/*D* = 2).

Figure 2 shows an example of the mesh and boundary conditions for the 3D stability analysis of a circular tunnel face in *C*/*D* = 2. The adaptive meshing method improve the solution accuracy as the mesh density is the greatest in zones with significant plastic shear strains, which is achieved by gradually optimizing the mesh size in zones with significant plastic shear strains. So, an automatically adaptive meshing method was employed herein, and four iterations of adaptive meshing with the initial number of elements increasing from 3000 to 10,000 were used in all analyses based on Shiau and Al-Asadi’s work^56^. In addition, using the load multiplier method in FELA, the rigorous UB and LB solutions of the limit pressure can be easily calculated.

## Results and discussions

### Load factor (σ_s_ − σ_t_)/c_u0_

For tunnel face stability analysis in purely cohesive soils, there are seven calculation parameters: cover depth *C*, tunnel diameter *D*, support pressure *σ*_*t*_, surface surcharge *σ*_*s*_, unit weight of soil *γ*, undrained cohesion of soil at ground surface *c*_*u0*_, and variation gradient of undrained cohesion *ρ*. According to previous studies, four dimensionless parameters were often used in undrained stability analysis^41,42^: cover depth ratio *C*/*D*, overburden stress factor *γD*/*c*_*u0*_, linear strength gradient ratio *ρD/c*_*u0*_, and load factor (*σ*_*s*_ − *σ*_*t*_)/*c*_*u0*_. The latter parameter is considered as a dependent parameter, and the other three parameters are regarded as independent parameters.

**Figure 3:**
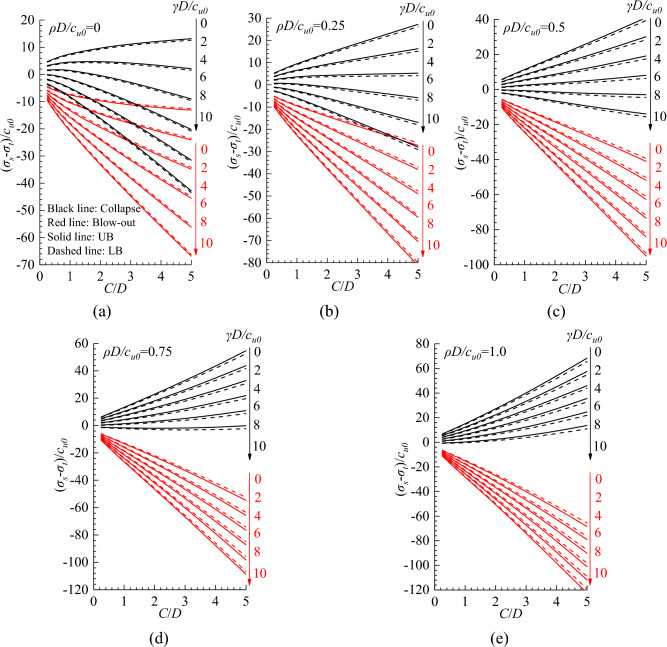
Relationship between (*σ*_*s*_ − *σ*_*t*_)/*c*_*u0*_, *C*/*D* and *γD*/*c*_*u0*_ for *ρD/c*_*u0*_ values of (**a**) 0, (**b**) 0.25, (**c**) 0.5, (**d**) 0.75 and (**e**) 1.0.

Nine hundred and sixty limit analyses were conducted to investigate this problem, in which *C*/*D* ranges from 0.25 to 5, *γD*/*c*_*u0*_ ranges from 0 to 10, and *ρD/c*_*u0*_ ranges from 0 to 1. These values were selected to cover a wide range of practical engineering conditions. Figure 3 and Table A1 shows the UB and LB solutions of (*σ*_*s*_ − *σ*_*t*_)/*c*_*u0*_ with different *C*/*D*, *γD*/*c*_*u0*_, and *ρD/c*_*u0*_. It can be observed that, in the collapse case, the value of (*σ*_*s*_ − *σ*_*t*_)/*c*_*u0*_ linearly decreases with increasing *γD*/*c*_*u0*_ and linearly increases with increasing *ρD/c*_*u0*_. However, in the blow-out case, the value of (*σ*_*s*_ − *σ*_*t*_)/*c*_*u0*_ linearly decreases with increasing *γD*/*c*_*u0*_ and *ρD/c*_*u0*_. This because under undrained conditions, the strength of soil is mainly determined by the undrained cohesion *c*_u_, while the friction angle of soil has no effect on the strength. And these findings align well with existing models^41,42^. Besides, the relationship between (*σ*_*s*_ − *σ*_*t*_)/*c*_*u0*_ and *C*/*D* are nonlinear in both the collapse and blow-out cases.

### Failure mechanism

**Figure 4:**
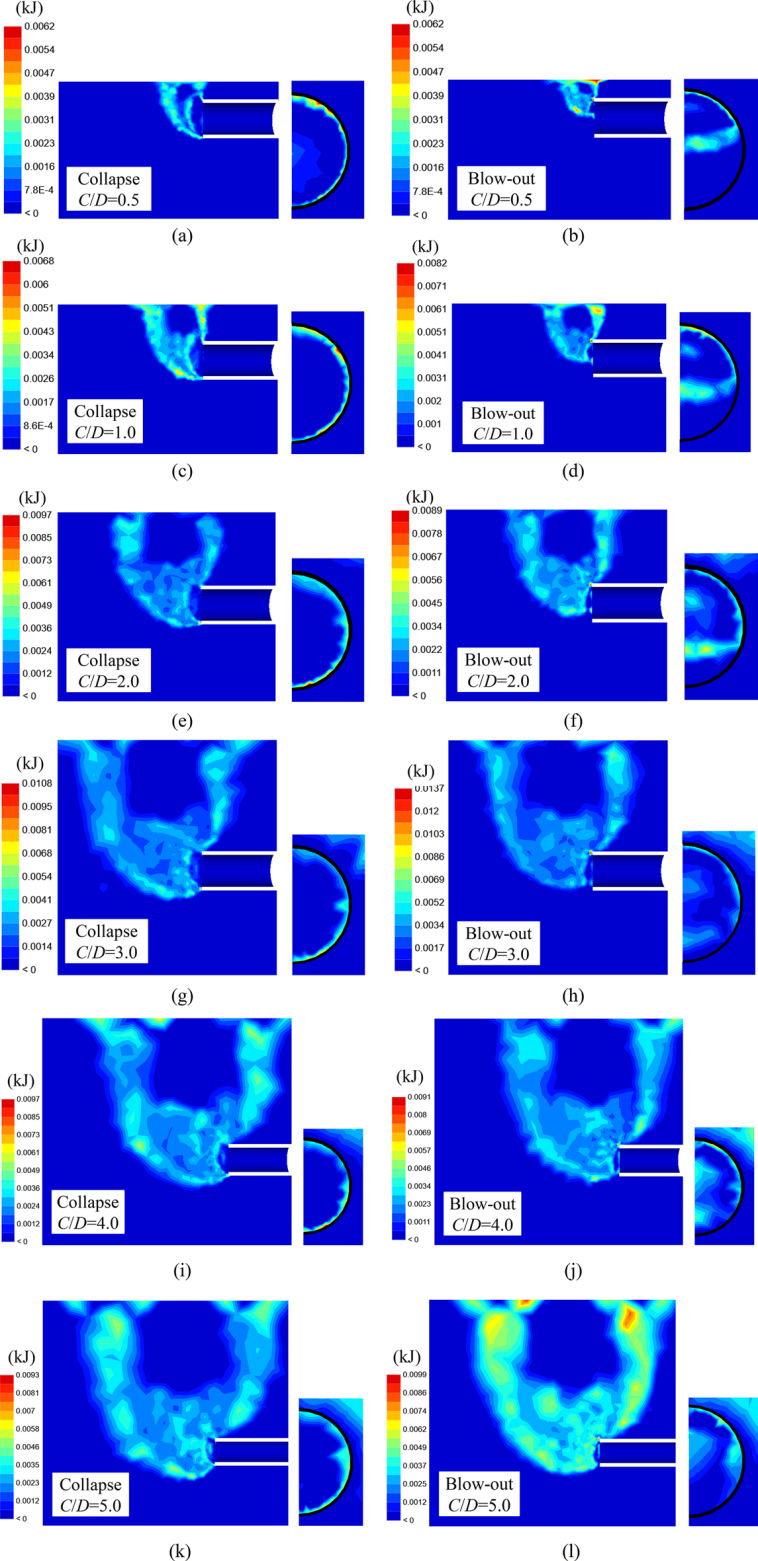
Shear dissipation contours in limit states of tunnel face collapse and blow-out.

Figure 4a–l show the shear dissipation contours in limit states of tunnel face collapse and blow-out for *C*/*D* = 0.25–3.0, which were output from FELA. The soil parameters *γD*/*c*_u0_ = 8 and *ρD*/*c*_u0_ = 0 were selected due to the limited space of the publication. Note that, in each figure, the left side is the lateral view of the tunnel, and the right side is the front view of the tunnel face. Apparently, the failure mechanisms in the collapse and blow-out cases have both similarities and differences.

In the collapse case, the failure zone in front of the tunnel face resembles a chimney-like mechanism, which complies with the existing numerical and experimental results^37,50^. In addition, the failure mechanism stretches out from the tunnel invert and then propagates to the ground surface, and the whole surface of the face is at failure. This kind of failure mechanism is called the global failure mechanism. Similar failure mechanisms have been employed by many authors in the establishment of analytical models.

In the blow-out case, the failure zone in front of the tunnel face is also a chimney-like mechanism. However, it is worth noting that for *C*/*D* < 2.0, the failure mechanism starts from a position above the tunnel invent instead of the tunnel invert, and only the upper part of the tunnel face is concerned by failure. This failure mechanism can be called the partial failure mechanism. Besides, the global failure mechanism can be observed in *C*/*D* > 2.0. This phenomenon indicates that in the blow-out case, there exists a gradual evolution process from partial failure to global failure within the tunnel face with increasing *C*/*D*. This partial failure phenomenon has been reported by many authors^12,52,53^. Still, the existing analytical models for tunnel face stability analysis in purely cohesive soils do not consider this mechanism, which may lead to inaccurate calculation results.

## New design equations

In practical engineering, it is necessary to evaluate the stability of the tunnel face quickly and accurately. However, numerical simulation usually requires a high-performance computer to achieve high computational efficiency, while analytical models are not rigorous enough owing to some essential assumptions (especially the failure mechanism). For the sake of application, two practical equations are presented based on the calculation results listed in Table A1 using the nonlinear regression analysis, as shown in Eqs. (1a) and (1b).

1a $$\frac{{\sigma_{s} - \sigma_{t} }}{{c_{u0} }} = a_{1} \left( \frac{C}{D} \right)^{{a_{2} }} + a_{3} \left( \frac{C}{D} \right)^{{a_{4} }} \left( {\frac{\rho D}{{c_{u0} }}} \right) - \left( {a_{5} + a_{6} \frac{C}{D}} \right)\left( {\frac{\gamma D}{{c_{u0} }}} \right){\text{ for collapse}}$$

1b $$\frac{{\sigma_{s} - \sigma_{t} }}{{c_{u0} }} = - a_{1} \left( \frac{C}{D} \right)^{{a_{2} }} - a_{3} \left( \frac{C}{D} \right)^{{a_{4} }} \left( {\frac{\rho D}{{c_{u0} }}} \right) - \left( {a_{5} + a_{6} \frac{C}{D}} \right)\left( {\frac{\gamma D}{{c_{u0} }}} \right){\text{ for blow-out}}$$

where coefficients *a*_*1*_-*a*_*6*_ for different conditions are listed in Table 1.

**Table 1:** Values of coefficients *a*_*1*_–*a*_*6*_.

Coefficient	Collapse		Blow-out	
	UB	LB	UB	LB
*a*_1_	7.7966	7.4695	7.8940	7.5667
*a*_2_	0.3173	0.3162	0.3271	0.3279
*a*_3_	7.5605	7.2994	7.4105	7.1867
*a*_4_	1.2398	1.2376	1.2458	1.2395
*a*_5_	0.5333	0.5363	0.2643	0.2570
*a*_6_	0.9940	0.9942	1.0319	1.0318

**Figure 5:**
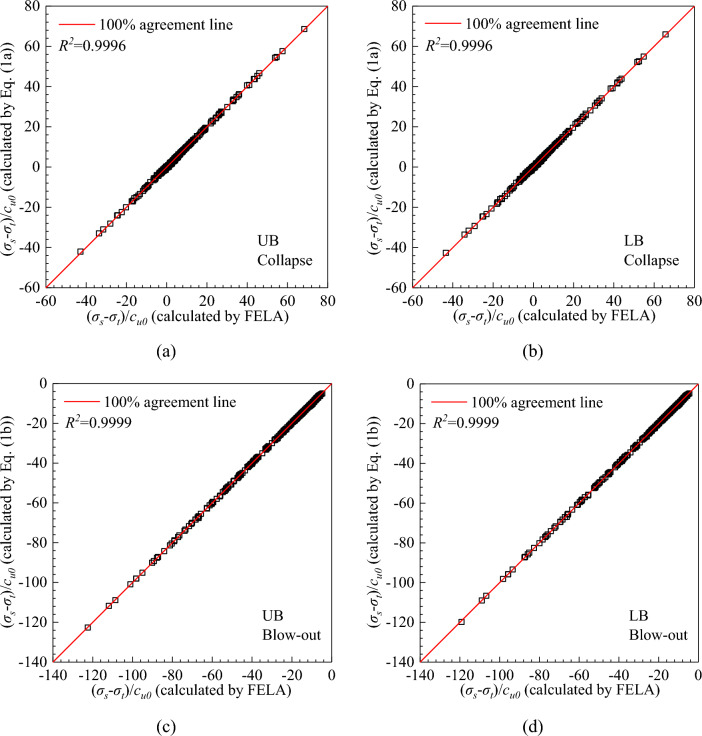
Comparisons of (*σ*_*s*_ − *σ*_*t*_)/*c*_*u0*_ between Eq. (1a), Eq. (1b) and FELA.

As shown in Fig. 5a–d, the results calculated by Eqs. (1a) and (1b) are compared with those from FELA. As expected, these two methods are in excellent agreement with a coefficient of determination (*R*^2^) of 99.96% (in the collapse case) and 99.99% (in the blow-out case).

Generally, the stability of a tunnel face is evaluated by the limit support pressure. Therefore, the equations that can be employed to calculate the UB and LB solutions of limit collapse and blow-out pressures are presented by adjusting Eqs. (1a) and (1b), as follows:

2a $$\sigma_{t} = \sigma_{s} + \gamma DN_{\gamma } - c_{u0} N_{c0} - \rho DN_{c\rho } {\text{ for collapse}}$$

2b $$\sigma_{t} = \sigma_{s} + \gamma DN_{\gamma } + c_{u0} N_{c0} + \rho DN_{c\rho } {\text{ for blow-out}}$$

where *N*_*c0*_, *N*_*cρ*_, and *N*_*γ*_ are the stability factors that respectively represent the effect of undrained cohesion *c*_*u0*_, variation gradient of undrained cohesion *ρ* and unit weight of soil *γ* on tunnel face stability:

2c $$N_{c0} = a_{1} \left( \frac{C}{D} \right)^{{a_{2} }}$$

2d $$N_{c\rho } = a_{3} \left( \frac{C}{D} \right)^{{a_{4} }}$$

2e $$N_{\gamma } = a_{5} + a_{6} \frac{C}{D}$$

In addition, a traditional method to empirically assess the stability of a tunnel face in purely cohesive soils is based on the stability number *N* that proposed by Broms and Bennermark^38^, as shown in Eq. (3).

3 $$N = \frac{{\sigma_{s} + \left( {C + {D \mathord{\left/ {\vphantom {D 2}} \right. \kern-0pt} 2}} \right)\gamma - \sigma_{t} }}{{c_{u0} }}$$

Substituting Eqs. (2a) and (2b) into Eq. (3) results in two equations of stability number *N*:

4a $$N = \frac{{\left( {C + {D \mathord{\left/ {\vphantom {D 2}} \right. \kern-0pt} 2}} \right)\gamma - \gamma DN_{\gamma } { + }c_{u0} N_{c0} { + }\rho DN_{c\rho } }}{{c_{u0} }}{\text{ for collapse}}$$

4b $$N = \frac{{\left( {C + {D \mathord{\left/ {\vphantom {D 2}} \right. \kern-0pt} 2}} \right)\gamma - \gamma DN_{\gamma } - c_{u0} N_{c0} - \rho DN_{c\rho } }}{{c_{u0} }}{\text{ for blow-out}}$$

## Comparisons with previous studies

To verify the present equations, the calculation results from proposed equations are compared with those from previous studies. These include three terms: stability number *N*, stability factors *N*_*c0*_, *N*_*cρ*_, *N*_*γ*_, and limit support pressure *σ*_*t*_.

### Comparisons of stability number *N*

**Figure 6:**
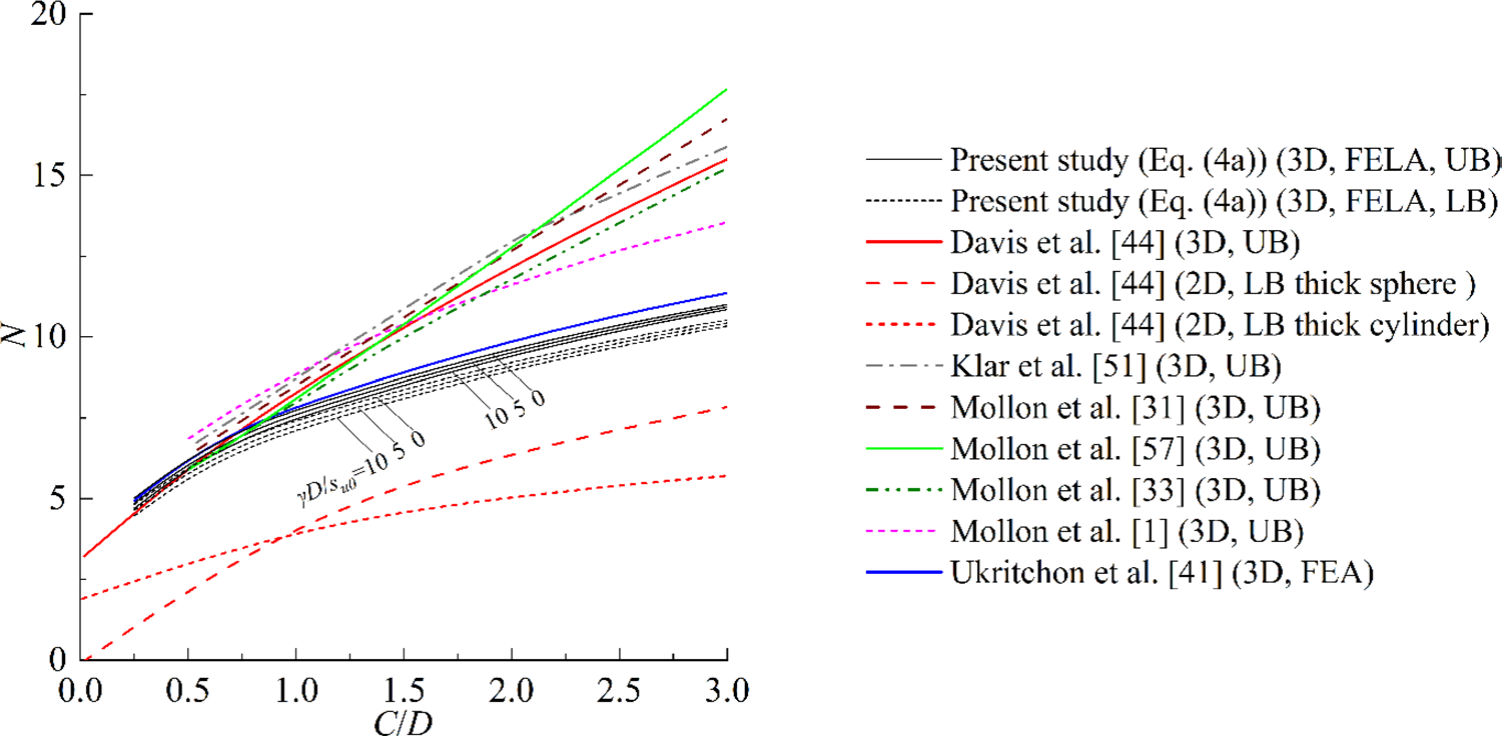
Comparisons of stability number *N* between this study and previous studies.

The ground is assumed to be homogeneous in most previous studies, therefore, *ρ* equals 0 in this section for the sake of comparison with existing methods. Since the stability factor *N*_*γ*_ in Eq. (2e) is not equal to (*C*/*D* + 0.5), the value of stability number *N* dependents on *γD*/*c*_*u0*_. To consider the effect of *γD*/*c*_*u0*_, three values of *γD*/*c*_*u0*_ = 0, 5, and 10 are considered in Fig. 6. Note that the equations proposed by Ukritchon et al.^41^ were derived from the results of FEM, and the analytical models^1,31,33,44,46,57^ were established based on the upper bound theory of the limit analysis method. Figure 6 shows that the effect of *γD*/*c*_*u0*_ on stability number *N* is not apparent, and the equations established in this study make a significant improvement over existing methods. This conclusion can be explained as that the analytical models need to presuppose a failure mechanism, but the failure mechanism employed in these methods maybe not the optimal one; in FEA, the limit support pressure is obtained by the so-called stress-controlled approach instead of rigorous analysis. Noticing that the LB solutions proposed by Davis et al.^44^ are significantly lower than other methods, because these solutions were based on the 2D plane strain condition.

### Comparisons of stability factors *N*_*c0*_, *N*_*cρ*_ and *N*_*γ*_

**Figure 7:**
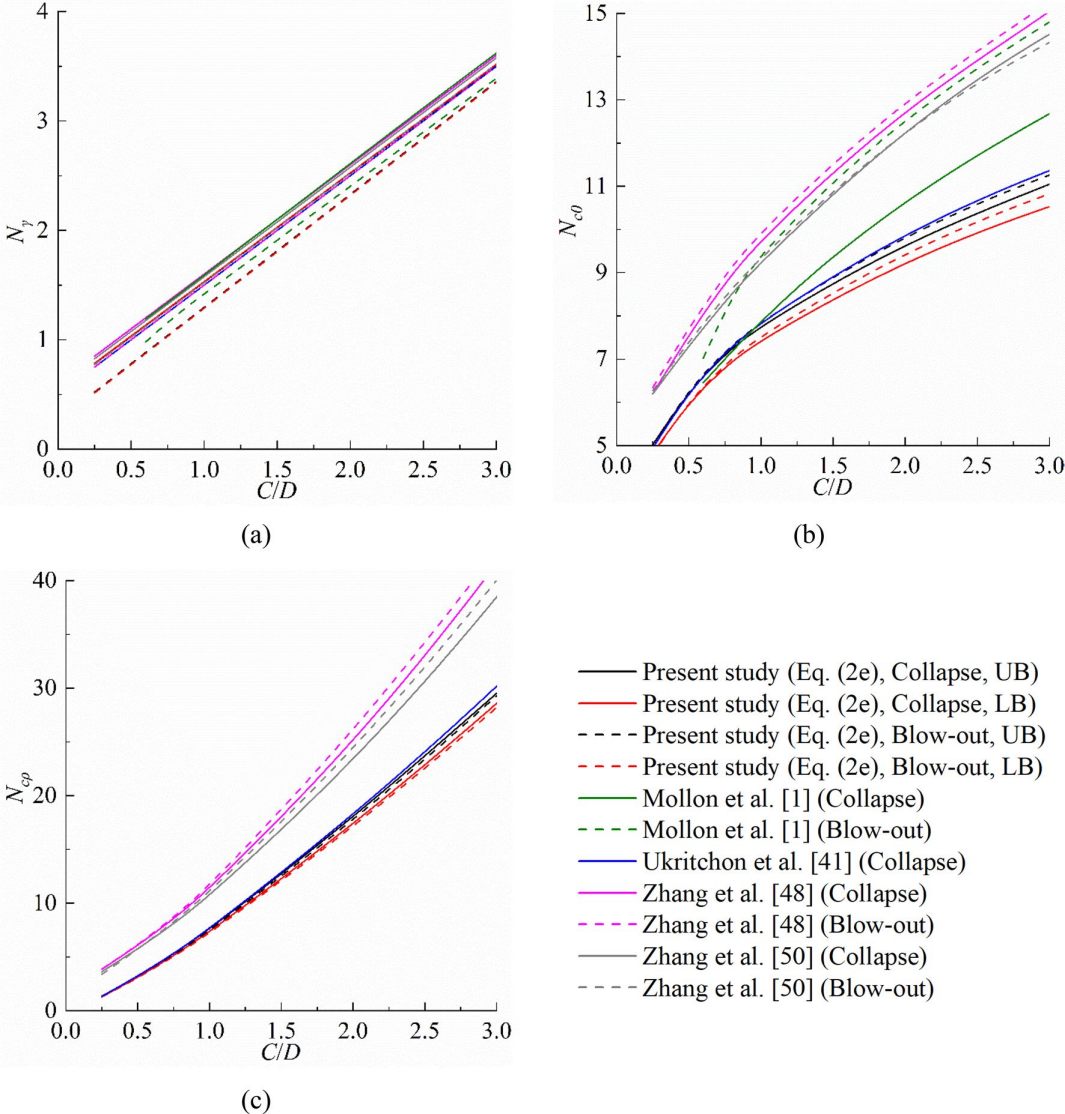
Comparisons of the stability factors between this study and previous studies: (**a**) *N*_*γ*_, (**b**) *N*_*c0*_ and (**c**) *N*_*cρ*_.

Figure 7a–c show the stability factors *N*_*c0*_*, N*_*cρ*_ and *N*_*γ*_ of tunnel face collapse and blow-out obtained by several methods for different overburden ratios *C*/*D*. For all methods, the relationship between *N*_*γ*_ and *C*/*D* is linear, while *N*_*c0*_ and *N*_*cρ*_ increase nonlinearly with the increase of *C*/*D*. As shown in Fig. 7a, the results of *N*_*γ*_ calculated by all methods are in close agreement. Then, it can be observed in Fig. 7b,c that the analytical models^1,47,50^ significantly overestimate *N*_*c0*_ and *N*_*cρ*_, and the equations proposed by Ukritchon et al.^41^ provide slightly larger estimates on *N*_*c0*_ and *N*_*cρ*_.

### Comparisons of limit support pressure *σ*_*t*_

**Figure 8:**
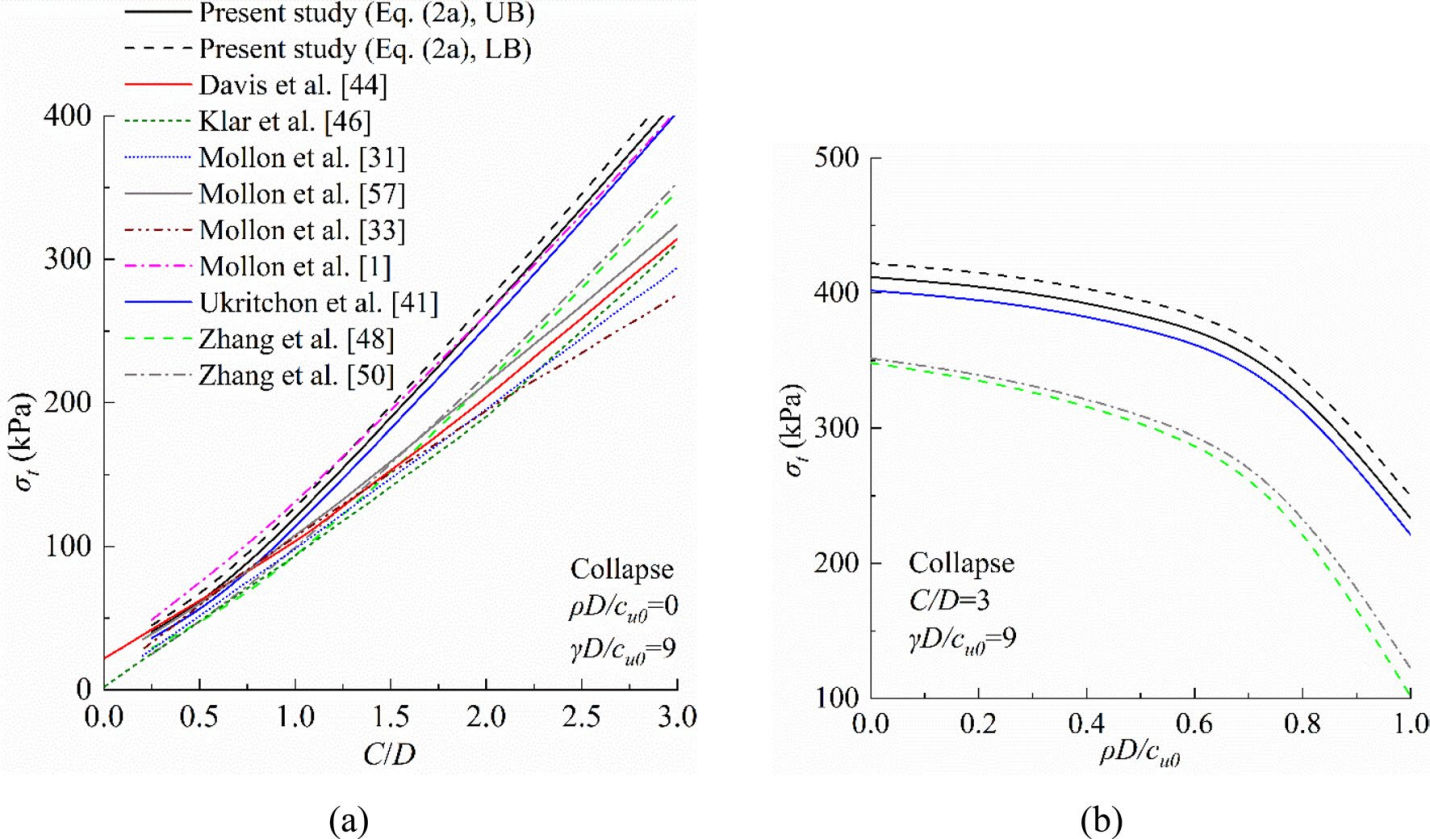
Comparisons of collapse pressure between this study and previous studies: (**a**) *ρD/c*_*u0*_ = 0 and (**b**) *ρD/c*_*u0*_ > 0.

**Figure 9:**
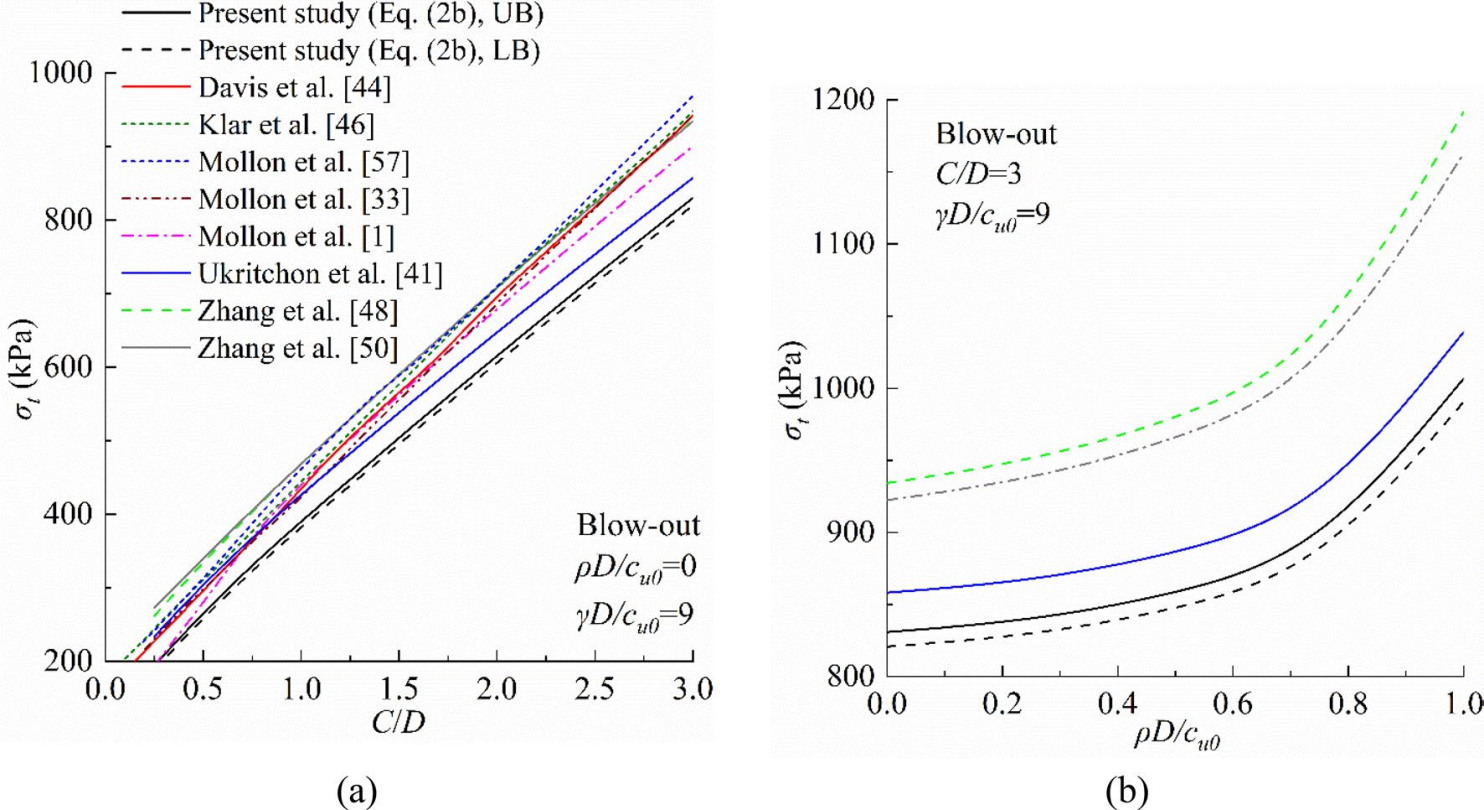
Comparisons of limit blow-out pressure between this study and previous studies: (**a**) *ρD/c*_*u0*_ = 0 and (**b**) *ρD/c*_*u0*_ > 0.

Figures 8 and 9 present the comparison in terms of the limit collapse and blow-out pressures for a tunnel of *D* = 10 m in homogeneous (*ρD/c*_*u0*_ = 0) and nonhomogeneous (*ρD/c*_*u0*_ > 0) soils with a unit weight *γ* = 18 kN/m^3^. It is evident that the present study provides the best result, because the limit collapse pressure calculated by Eq. (2a) is the largest of all methods, while the limit blow-out pressure calculated by Eq. (2b) is the smallest of all methods^1^. This because the partial failure mechanism is not considered in these methods. To be specific, the limit collapse pressure calculated by Mollon et al.^1^ agrees well with those from Eq. (2a), but the agreement decreases in the blow-out case. Besides, the inhomogeneity of soil is neglected in this model, which may limit its application. Meanwhile, other analytical models significantly underestimate the limit collapse pressure and overestimate the limit blow-out pressure, which indicates that the failure mechanism employed in these models can be further improved. As shown in Figs. 8b and 9b, the inhomogeneity of soil has a significant influence on tunnel face stability. The limit collapse pressure decreases with the increases of *ρD/c*_*u0*_, while the limit blow-out pressure increases with increasing *ρD/c*_*u0*_. Therefore, the inhomogeneity of soil cannot be ignored in practical engineering. Also, in the collapse case, the results obtained by the equations proposed by Ukritchon et al.^41^ slightly underestimate the limit support pressure. However, in the blow-out case, the difference between Eq. (2b) and Ukritchon et al.’s equations are visible. Additionally, as shown in Fig. 9, because the partial failure mechanism is considered in this paper, the results of the limit blow-out pressure is smaller than those of other models. So, if this partial failure mechanism is not considered, the limit blow-out pressure of the tunnel face will be overestimated.

## Application

A proposed Yakang highway tunnel has a diameter (*D*) of 10 m and a cover depth (*C*) of 5 m. The soil is found to be purely cohesive soil with a unit weight (γ) of 18 kN/m^3^, undrained cohesion at ground (*c*_*u0*_) of 20 kPa, variation gradient of undrained cohesion (*ρ*) of 0.4. Determine the limit support pressure (*σ*_t_) when the surcharge pressure (*σ*_s_) is zero. According to Eqs. (2a)–(2e) and Table 1, the following results can be obtained, as shown in Table 2.

**Table 2:** Calculation results.

Coefficient	Collapse		Blow-out	
	UB	LB	UB	LB
*N*_*c0*_	6.257	5.999	6.293	6.021
*N*_*cρ*_	3.201	3.096	3.125	3.044
*N*_*γ*_	1.030	1.033	0.780	0.773
*σ*_t_ (kPa)	406.37	416.71	836.22	825.98

The comparisons of limit pressures between this study and previous studies are listed in Table 3. It is evident that the present study provides the best result, because the limit collapse pressure calculated by this method is the largest of all methods, while the limit blow-out pressure calculated by this method is the smallest of all methods.

**Table 3:** Comparisons of limit pressures between this study and previous studies.

Method	Limit collapse pressure	Limit blow-out pressure
Proposed method	406.37	836.22
Ukritchon et al.^48^	396.42	863.58
Zhang et al. ^56^	332.70	949.69
Zhang et al.^58^	337.13	935.95

## Conclusions

To study the face stability of circular tunnels in purely cohesive soils with linearly increasing strength, numerical simulations were performed employing the 3D FELA. A wide range of ground conditions was selected to cover a broad scope of practical application, and the rigorous UB and LB solutions of load factor (*σ*_*s*_ − *σ*_*t*_)/*c*_*u0*_ were calculated. Using the nonlinear regression analysis method, a series of equations of the limit support pressure *σ*_*t*_ and the stability number *N* in both the collapse and blow-out cases were proposed based on 960 numerical results. The following conclusions are drawn:

1. A nonlinear relationship between (*σ*_*s*_ − *σ*_*t*_)/*c*_*u0*_ and *C*/*D* was found in both the collapse and blow-out cases. Moreover, in the collapse case, the value of (*σ*_*s*_ − *σ*_*t*_)/*c*_*u0*_ linearly decreases with increasing *γD*/*c*_*u0*_ and linearly increase with increasing *ρD/c*_*u0*_. In the blow-out case, the value of (*σ*_*s*_ − *σ*_*t*_)/*c*_*u0*_ linearly decreases with increasing *γD*/*c*_*u0*_ and *ρD/c*_*u0*_.
2. In both the collapse and blow-out cases, the failure zone in front of the tunnel face is chimney-like. In the collapse case, the failure mechanism is global and stretches out from the tunnel invert. Also, the entire surface of the face is at failure. In the blow-out case, the failure mechanism is partial in *C*/*D* < 2.0, which starts from a position above the tunnel invent instead of the tunnel invert, and the failure zone includes only the upper part of the tunnel face; when *C*/*D* > 2, the failure mechanism is also global.
3. The comparisons showed that the proposed equations make an improvement over existing methods, and these equations provide a quick and accurate estimation on tunnel face stability in purely cohesive soils.
4. However, our results are applicable to *C*/*D* = 0.25–5, which can already cover most practical projects. However, in an extreme larger burial depth condition, our results may be invalid, and more calculations need to be performed further. In the future research, it is suggested to further establish a more widely applicable theoretical analytical model for tunnel stability analysis in purely cohesive soils based on the research results of this paper.

## Data availability

All data generated or analysed during this study are included in this published article.

### Supplementary Information

Supplementary Information.

## Abbreviations

*a*_*1*_-*a*_*6*_: Calculation coefficients
*C*: Cover depth
*D*: Tunnel diameter
DEM: Discrete element method
FEM: Finite element method
FDM: Finite difference method
FELA: Finite element limit analysis
LB: Lower bound
UB: Upper bound
*R*^2^: Coefficient of determination
*c*_*u*_: Undrained cohesion of soil
*c*_*u0*_: Undrained cohesion of soil at ground surface
*ρ*: Variation gradient of undrained cohesion
*σ*_*s*_: Surface surcharge
*σ*_*t*_: Support pressure
*γ*: Unit weight of soil
*x*,* y*: Coordinates of the Cartesian system
*N*_*c0*_, *N*_*c*__*ρ*_, *N*_*γ*_: Stability factors
*N*: Stability number
