# Supplementary material for: Numerical analyses for three-dimensional face stability of circular tunnels in purely cohesive soils with linearly increasing strength
Source: Sci Rep. 2024 Mar 13;14:6101. doi: 10.1038/s41598-023-49065-6 (PMC10937965; doi:10.1038/s41598-023-49065-6)
Supplement: Supplementary file 1 — Supplementary Information. [file 41598_2023_49065_MOESM1_ESM.docx]

# Appendix A Calculation results output from FELA

Table A1 UB and LB solutions of (*σ_s_*-*σ_t_*)/*c_u0_* for different *C*/*D*, *γD*/*c_u0_* and *ρD/c_u0_* in the collapse and blow-out cases.

| Collapse | | | | | | | | | | | | | |
| --- | --- | --- | --- | --- | --- | --- | --- | --- | --- | --- | --- | --- | --- |
| *C*/*D* | *ρD*/*c_u0_* | *γD*/*c_u0_*=0 | | *γD*/*c_u0_*=2 | | *γD*/*c_u0_*=4 | | *γD*/*c_u0_*=6 | | *γD*/*c_u0_*=8 | | *γD*/*c_u0_*=10 | |
|  |  | UB | LB | UB | LB | UB | LB | UB | LB | UB | LB | UB | LB |
| 0.25 | 0 | 4.726 | 4.529 | 3.256 | 3.005 | 1.638 | 1.427 | -0.021 | -0.230 | -1.728 | -1.980 | -3.436 | -3.731 |
| 0.25 | 0.25 | 5.274 | 5.071 | 3.876 | 3.660 | 2.351 | 2.086 | 0.706 | 0.454 | -0.994 | -1.204 | -2.697 | -2.938 |
| 0.25 | 0.5 | 5.787 | 5.567 | 4.469 | 4.239 | 2.970 | 2.728 | 1.386 | 1.129 | -0.228 | -0.516 | -1.900 | -2.221 |
| 0.25 | 0.75 | 6.274 | 6.019 | 5.022 | 4.749 | 3.597 | 3.333 | 2.071 | 1.771 | 0.431 | 0.157 | -1.208 | -1.500 |
| 0.25 | 1 | 6.731 | 6.473 | 5.562 | 5.303 | 4.190 | 3.927 | 2.679 | 2.402 | 1.093 | 0.806 | -0.512 | -0.825 |
| 0.5 | 0 | 5.853 | 5.618 | 3.862 | 3.609 | 1.752 | 1.513 | -0.384 | -0.661 | -2.633 | -2.920 | -4.882 | -5.180 |
| 0.5 | 0.25 | 6.821 | 6.536 | 4.865 | 4.581 | 2.818 | 2.529 | 0.697 | 0.405 | -1.470 | -1.763 | -3.676 | -3.974 |
| 0.5 | 0.5 | 7.752 | 7.438 | 5.825 | 5.517 | 3.830 | 3.501 | 1.730 | 1.413 | -0.388 | -0.719 | -2.538 | -2.867 |
| 0.5 | 0.75 | 8.624 | 8.292 | 6.759 | 6.421 | 4.796 | 4.439 | 2.748 | 2.398 | 0.654 | 0.295 | -1.471 | -1.842 |
| 0.5 | 1 | 9.494 | 9.146 | 7.689 | 7.323 | 5.756 | 5.376 | 3.769 | 3.354 | 1.699 | 1.293 | -0.407 | -0.827 |
| 0.75 | 0 | 6.824 | 6.530 | 4.315 | 4.024 | 1.716 | 1.436 | -0.960 | -1.238 | -3.697 | -3.993 | -6.433 | -6.747 |
| 0.75 | 0.25 | 8.223 | 7.867 | 5.773 | 5.447 | 3.228 | 2.897 | 0.630 | 0.289 | -2.022 | -2.491 | -4.756 | -5.092 |
| 0.75 | 0.5 | 9.593 | 9.203 | 7.155 | 6.781 | 4.673 | 4.286 | 2.112 | 1.729 | -0.486 | -0.904 | -3.142 | -3.551 |
| 0.75 | 0.75 | 10.922 | 10.493 | 8.554 | 8.105 | 6.090 | 5.629 | 3.530 | 3.108 | 0.986 | 0.531 | -1.605 | -2.090 |
| 0.75 | 1 | 12.256 | 11.783 | 9.920 | 9.379 | 7.486 | 6.977 | 5.000 | 4.486 | 2.443 | 1.937 | -0.148 | -0.666 |
| 1 | 0 | 7.609 | 7.271 | 4.624 | 4.299 | 1.510 | 1.202 | -1.679 | -1.976 | -4.920 | -5.257 | -8.162 | -8.538 |
| 1 | 0.25 | 9.526 | 9.131 | 6.584 | 6.198 | 3.560 | 3.169 | 0.471 | 0.069 | -2.682 | -3.095 | -5.916 | -6.297 |
| 1 | 0.5 | 11.405 | 10.942 | 8.488 | 8.015 | 5.488 | 5.048 | 2.441 | 1.993 | -0.650 | -1.113 | -3.785 | -4.259 |
| 1 | 0.75 | 13.220 | 12.721 | 10.347 | 9.795 | 7.397 | 6.855 | 4.388 | 3.854 | 1.345 | 0.788 | -1.756 | -2.314 |
| 1 | 1 | 15.051 | 14.476 | 12.219 | 11.606 | 9.286 | 8.669 | 6.278 | 5.693 | 3.297 | 2.655 | 0.186 | -0.444 |
| 2 | 0 | 9.801 | 9.410 | 4.819 | 4.405 | -0.298 | -0.707 | -5.493 | -5.893 | -10.777 | -11.184 | -16.060 | -16.476 |
| 2 | 0.25 | 14.242 | 13.647 | 9.307 | 8.710 | 4.274 | 3.691 | -0.812 | -1.393 | -5.915 | -6.504 | -11.111 | -11.704 |
| 2 | 0.5 | 18.532 | 17.798 | 13.629 | 12.889 | 8.656 | 7.930 | 3.635 | 2.905 | -1.464 | -2.177 | -6.558 | -7.287 |
| 2 | 0.75 | 22.817 | 21.913 | 17.931 | 17.008 | 12.978 | 12.071 | 7.953 | 7.032 | 2.950 | 2.038 | -2.093 | -3.017 |
| 2 | 1 | 27.042 | 26.003 | 22.144 | 20.986 | 17.205 | 16.176 | 12.254 | 11.208 | 7.243 | 6.217 | 2.186 | 1.140 |
| 3 | 0 | 11.226 | 10.755 | 4.245 | 3.759 | -2.863 | -3.343 | -10.067 | -10.551 | -17.344 | -17.889 | -24.620 | -25.227 |
| 3 | 0.25 | 18.634 | 17.856 | 11.657 | 10.887 | 4.663 | 3.884 | -2.396 | -3.203 | -9.591 | -10.272 | -16.676 | -17.433 |
| 3 | 0.5 | 25.806 | 24.772 | 18.885 | 17.829 | 11.908 | 10.859 | 4.872 | 3.841 | -2.194 | -3.193 | -9.360 | -10.286 |
| 3 | 0.75 | 32.900 | 31.609 | 26.027 | 24.719 | 19.054 | 17.747 | 12.116 | 10.750 | 5.084 | 3.758 | -1.886 | -3.325 |
| 3 | 1 | 39.927 | 38.434 | 33.167 | 31.605 | 26.237 | 24.646 | 19.281 | 17.653 | 12.262 | 10.662 | 5.290 | 3.693 |
| 4 | 0 | 12.291 | 11.773 | 3.281 | 2.763 | -5.822 | -6.366 | -15.031 | -15.576 | -24.320 | -24.867 | -33.609 | -34.158 |
| 4 | 0.25 | 22.917 | 21.973 | 13.967 | 13.002 | 4.936 | 3.973 | -4.094 | -5.034 | -13.302 | -14.152 | -22.344 | -23.286 |
| 4 | 0.5 | 33.273 | 31.938 | 24.343 | 23.056 | 15.406 | 14.028 | 6.415 | 5.022 | -2.623 | -4.007 | -11.732 | -13.021 |
| 4 | 0.75 | 43.322 | 41.787 | 34.680 | 32.925 | 25.741 | 24.002 | 16.736 | 15.007 | 7.719 | 5.970 | -1.267 | -3.000 |
| 4 | 1 | 53.851 | 51.649 | 45.023 | 42.888 | 35.990 | 33.939 | 27.113 | 24.935 | 18.139 | 15.968 | 9.052 | 6.923 |
| 5 | 0 | 13.137 | 12.579 | 2.121 | 1.543 | -9.019 | -9.589 | -20.226 | -20.796 | -31.524 | -32.125 | -42.822 | -43.454 |
| 5 | 0.25 | 27.193 | 26.065 | 16.244 | 15.093 | 5.266 | 4.125 | -5.785 | -6.920 | -16.918 | -18.039 | -27.996 | -29.140 |
| 5 | 0.5 | 40.998 | 39.365 | 30.110 | 28.360 | 19.130 | 17.406 | 8.071 | 6.375 | -2.964 | -4.619 | -14.015 | -15.626 |
| 5 | 0.75 | 54.641 | 52.481 | 43.732 | 41.651 | 32.855 | 30.649 | 21.889 | 19.646 | 10.909 | 8.723 | -0.130 | -2.352 |
| 5 | 1 | 68.361 | 65.687 | 57.558 | 54.827 | 46.000 | 43.818 | 35.657 | 32.804 | 24.667 | 21.892 | 13.677 | 10.913 |
| Blow-out | | | | | | | | | | | | | |
| *C*/*D* | *ρD/c_u0_* | *γD*/*c_u0_*=0 | | *γD*/*c_u0_*=2 | | *γD*/*c_u0_*=4 | | *γD*/*c_u0_*=6 | | *γD*/*c_u0_*=8 | | *γD*/*c_u0_*=10 | |
|  |  | UB | LB | UB | LB | UB | LB | UB | LB | UB | LB | UB | LB |
| 0.25 | 0 | -4.721 | -4.521 | -5.981 | -5.769 | -7.024 | -6.796 | -7.935 | -7.699 | -8.777 | -8.521 | -9.619 | -9.344 |
| 0.25 | 0.25 | -5.269 | -5.067 | -6.437 | -6.204 | -7.445 | -7.203 | -8.335 | -8.074 | -9.193 | -8.913 | -9.981 | -9.687 |
| 0.25 | 0.5 | -5.782 | -5.553 | -6.882 | -6.655 | -7.867 | -7.589 | -8.767 | -8.463 | -9.615 | -9.279 | -10.373 | -10.024 |
| 0.25 | 0.75 | -6.263 | -6.024 | -7.330 | -7.069 | -8.302 | -8.007 | -9.195 | -8.856 | -9.984 | -9.653 | -10.760 | -10.402 |
| 0.25 | 1 | -6.724 | -6.465 | -7.769 | -7.481 | -8.659 | -8.381 | -9.544 | -9.217 | -10.388 | -10.011 | -11.162 | -10.788 |
| 0.5 | 0 | -5.868 | -5.621 | -7.724 | -7.489 | -9.402 | -9.138 | -10.934 | -10.664 | -12.379 | -12.075 | -13.823 | -13.485 |
| 0.5 | 0.25 | -6.821 | -6.537 | -8.616 | -8.341 | -10.257 | -9.977 | -11.785 | -11.464 | -13.227 | -12.885 | -14.563 | -14.240 |
| 0.5 | 0.5 | -7.724 | -7.437 | -9.494 | -9.185 | -11.098 | -10.781 | -12.631 | -12.275 | -14.062 | -13.697 | -15.425 | -15.051 |
| 0.5 | 0.75 | -8.629 | -8.285 | -10.344 | -10.024 | -11.920 | -11.596 | -13.449 | -13.077 | -14.879 | -14.489 | -16.242 | -15.845 |
| 0.5 | 1 | -9.505 | -9.151 | -11.186 | -10.816 | -12.741 | -12.388 | -14.276 | -13.868 | -15.652 | -15.223 | -17.093 | -16.651 |
| 0.75 | 0 | -6.820 | -6.530 | -9.208 | -8.919 | -11.382 | -11.136 | -13.503 | -13.213 | -15.500 | -15.174 | -17.496 | -17.136 |
| 0.75 | 0.25 | -8.189 | -7.895 | -10.562 | -10.234 | -12.775 | -12.430 | -14.851 | -14.516 | -16.872 | -16.471 | -18.791 | -18.361 |
| 0.75 | 0.5 | -9.577 | -9.203 | -11.923 | -11.489 | -14.017 | -13.715 | -16.182 | -15.767 | -18.201 | -17.770 | -20.153 | -19.687 |
| 0.75 | 0.75 | -10.925 | -10.493 | -13.138 | -12.798 | -15.391 | -14.955 | -17.490 | -17.045 | -19.476 | -19.028 | -21.449 | -20.975 |
| 0.75 | 1 | -12.276 | -11.783 | -14.369 | -14.041 | -16.673 | -16.208 | -18.806 | -18.269 | -20.675 | -20.248 | -22.784 | -22.223 |
| 1 | 0 | -7.614 | -7.289 | -10.491 | -10.165 | -13.258 | -12.913 | -15.850 | -15.518 | -18.378 | -18.047 | -20.907 | -20.576 |
| 1 | 0.25 | -9.528 | -9.141 | -12.380 | -12.008 | -15.128 | -14.707 | -17.789 | -17.344 | -20.324 | -19.894 | -22.799 | -22.322 |
| 1 | 0.5 | -11.391 | -10.938 | -14.187 | -13.781 | -16.959 | -16.495 | -19.591 | -19.139 | -22.209 | -21.681 | -24.507 | -24.154 |
| 1 | 0.75 | -13.239 | -12.712 | -16.067 | -15.521 | -18.785 | -18.265 | -21.430 | -20.922 | -24.040 | -23.476 | -26.555 | -25.951 |
| 1 | 1 | -15.055 | -14.462 | -17.753 | -17.294 | -20.576 | -19.948 | -23.291 | -22.649 | -25.685 | -25.164 | -28.308 | -27.681 |
| 2 | 0 | -9.812 | -9.404 | -14.705 | -14.273 | -19.491 | -19.052 | -24.169 | -23.714 | -28.720 | -28.281 | -33.272 | -32.849 |
| 2 | 0.25 | -14.238 | -13.638 | -19.079 | -18.496 | -23.920 | -23.306 | -28.589 | -27.978 | -33.258 | -32.641 | -37.858 | -37.224 |
| 2 | 0.5 | -18.528 | -17.818 | -23.441 | -22.653 | -28.191 | -27.403 | -32.957 | -31.623 | -37.642 | -36.857 | -42.323 | -41.346 |
| 2 | 0.75 | -22.789 | -21.861 | -27.475 | -26.735 | -32.433 | -31.528 | -37.185 | -36.020 | -41.880 | -40.988 | -46.555 | -45.643 |
| 2 | 1 | -27.083 | -25.959 | -31.750 | -30.763 | -36.747 | -35.573 | -41.460 | -40.438 | -46.181 | -44.961 | -50.434 | -49.713 |
| 3 | 0 | -11.251 | -10.768 | -18.104 | -17.641 | -24.917 | -24.407 | -31.642 | -31.106 | -38.229 | -37.666 | -44.816 | -44.227 |
| 3 | 0.25 | -18.621 | -17.850 | -25.505 | -24.717 | -32.247 | -31.523 | -39.063 | -38.269 | -45.780 | -44.775 | -52.414 | -51.578 |
| 3 | 0.5 | -25.829 | -24.719 | -32.697 | -31.594 | -39.545 | -38.442 | -46.332 | -45.223 | -53.071 | -51.923 | -59.679 | -58.656 |
| 3 | 0.75 | -32.794 | -31.574 | -39.771 | -38.521 | -46.640 | -45.253 | -53.068 | -51.916 | -60.111 | -58.891 | -66.667 | -65.518 |
| 3 | 1 | -40.027 | -38.453 | -46.877 | -45.302 | -53.638 | -52.065 | -60.336 | -58.841 | -67.319 | -65.613 | -73.913 | -72.449 |
| 4 | 0 | -12.292 | -11.782 | -21.219 | -20.672 | -30.011 | -29.473 | -38.714 | -38.143 | -47.324 | -46.746 | -55.934 | -55.349 |
| 4 | 0.25 | -22.889 | -21.935 | -31.803 | -30.850 | -40.601 | -39.611 | -49.309 | -48.440 | -57.741 | -57.145 | -66.879 | -65.878 |
| 4 | 0.5 | -33.207 | -31.897 | -42.135 | -40.821 | -50.922 | -49.642 | -59.675 | -58.487 | -68.332 | -67.150 | -77.175 | -75.848 |
| 4 | 0.75 | -43.562 | -41.913 | -52.265 | -50.777 | -61.285 | -59.651 | -70.152 | -68.311 | -78.970 | -77.067 | -87.745 | -85.833 |
| 4 | 1 | -53.766 | -51.724 | -62.623 | -60.612 | -71.661 | -69.546 | -80.368 | -78.368 | -89.295 | -87.216 | -98.153 | -95.694 |
| 5 | 0 | -13.144 | -12.595 | -24.061 | -23.491 | -34.832 | -34.168 | -45.548 | -44.893 | -56.179 | -55.541 | -66.810 | -66.189 |
| 5 | 0.25 | -27.227 | -26.080 | -38.084 | -36.981 | -48.986 | -47.744 | -59.506 | -58.606 | -70.470 | -69.387 | -81.228 | -79.950 |
| 5 | 0.5 | -41.001 | -39.360 | -51.821 | -50.227 | -62.712 | -61.012 | -73.461 | -71.836 | -84.116 | -82.694 | -95.048 | -93.448 |
| 5 | 0.75 | -54.711 | -52.490 | -65.685 | -63.449 | -76.487 | -74.214 | -87.210 | -85.077 | -98.190 | -95.701 | -108.646 | -106.681 |
| 5 | 1 | -68.423 | -65.622 | -79.075 | -76.491 | -90.239 | -87.471 | -101.110 | -98.245 | -111.925 | -108.967 | -122.516 | -119.137 |
